# Is the safety of herbal medicines for kidneys under question?

**Published:** 2013-07-01

**Authors:** Mohammad-Reza Ardalan, Mahmoud Rafieian-Kopaei

**Affiliations:** ^1^Chronic Kidney Disease Research Center, Tabriz University of Medical Sciences, Tabriz, Iran; ^2^Medical Plants Research Center, Shahrekord University of Medical Sciences, Shahrekord, Iran

**Keywords:** Adverse effects, Safety, Herbal toxicities, Medical plants

**Implication for health policy/practice/research/medical education:**

Herbal medicines are generally considered to be safe and effective agents. Therefore, people more and more turn to herbal medicine because they believe that plant remedies are free from undesirable side effects. However, medicinal plants can be toxic intrinsically or when taken in combination with other preparations. Herbal products are usually not evaluated for purity and consistency of active compounds, they also often contain contaminants. Furthermore, it is believed that if a drug is effective, it will not totally be safe and will have side effects. Therefore, herbal medicines as drugs may have side effects, too. More research on adverse reactions on available herbal preparations should be encouraged and public education on the good and bad effects of herbals need to be emphasized. Health care professionals should remain vigilant for potential interactions between herbals and prescription medications.

Nowadays, more and more people are turning away from conventional medicines, because they believe that ‘natural’ substances like medicinal plants are safer than synthetic substances. The increasing use of medicinal plants has resulted in concern about the efficacy and safety of herbal products (1).

It is estimated that 80% of the world population consume different forms of herbal products and the use of herbal medicines is growing. It is estimated that the usage of herbal products increases annually at a rate of 10-20% (2).

Despite the shortage of knowledge about the pharmacological efficacies and safety of herbals, sales of tested and untested preparations are on the rise. Of primary concern is the quality, safety and reliability of the products available in the marketplace, since prior to marketing safety and efficacy are not guaranteed and no monitoring of the potency or safety of the herbals is required. This is in contrast to the requirements for pharmaceutical products prior to registration (3).

Medicinal plants can be toxic intrinsically or when taken in combination with other preparations. Herbal products are usually not evaluated for purity and consistency of active compounds, they also often contain contaminants. Furthermore, it is believed that if a drug is effective, it will not totally be safe and will have side effects. Therefore, herbal medicines as drugs may have side effects, too. However, herbal medicines are generally considered to be safe and effective agents. Therefore, people more and more turn to herbal medicine because they believe that plant remedies are free from undesirable side effects (1).

Plants have important roles in the development of modern medicines. It is estimated that about 65% of modern medicines are directly or indirectly derived from plants. However, herbs are not non-toxic just because they are natural. Medicinal herbs contain powerful, pharmacologically active compounds. While some herbs appear to be fairly safe, all medicines should be used with caution. Although the safety beliefs of the population about the herbs are partially true, however, inclusion of incorrect and toxic species, allergens, insect parts, heavy metals such as mercury, lead and arsenic, whether intentional or by mistake, have been cited as the causes of herbal adverse reactions or toxicities (4).

Approximately 8% of hospital admissions in the US are due to side effects of synthetic drugs. At least one hundred thousand people die from these toxicities, each year. That means that at least three times as many people are killed in the US by pharmaceutical drugs as are killed by drunken drivers. Each year thousands of people die from supposedly “safe” over-the-counter remedies. Hospitalizations or deaths due to herbs are so rare that the United State National Poison Control Centers do not even have a category in their database for adverse reactions to medicinal plants. However, the increase in the use of herbal products means that there is more potential for adverse effects and drug interactions, particularly between herbal products and conventional drugs (5).

Therefore, more herbal toxicity and drug-herb interaction studies should be encouraged.

Public should also be made aware of the side effects of medicinal plants and patients should be asked about their use of herbal products in order to evaluate the potential interact with concurrent drugs.

The safety of herbal products is important because the majority of these products is self-prescribed and is used to treat chronic conditions. Otherwise, most patients consuming herbal preparations are not aware of the potential side effects. Therefore, these preparations may cause complications. Basically, people who consume medicinal products are be classified into four groups (4):

a. Those people who use herbal products for therapeutic purposes while dismissing the efficacy of modern medicines.
b. Those people who use herbal products more than conventional medicines for therapeutic purposes. These patients may do so only after realizing that modern medicine has nothing more to offer in treating their health problems.
c. Those who use modern medicines more than herbal products for therapeutic purposes. This group usually constitutes the majority of world population.
d. Those people who do not believe in the efficacy of herbal medicines and use only modern medicines. This group of people, unfortunately are unaware of the role of herbals in drug development.

The first reason for the use of herbals is the belief of people for maintenance of health or to treat certain ailments. The second reason is the relatively cheaper cost of herbal products which is important for the lower income group. The third reason for the use of herbals is that herbals are natural and this group of users believes that anything natural is safe (1).

The forth reason is the belief herbal products do not contain chemicals and that chemicals, are linked to more adverse effects and toxicity, and hence are more harmful.

It should be noted that the number of reports about the adverse effects of herbal products is now increasing due to increased use and increased awareness among the consumers and clinical practitioners. Patients consuming herbal preparations should be aware that herbs might cause toxic reactions. Certain groups of the population should be extra cautious as they are more susceptible to herbal adverse reactions or toxicities. They include pregnant and nursing women; some compounds in herbs can cross the placenta and are clearly linked to birth defects or other problems in newborns. Infants, children and elderly people are much more sensitive than adults to the effects of all medicines including herbs (3).

In conclusion, findings by many researchers have reinforced the idea that consumption of natural medicines may not be without risk. More research on adverse reactions on available herbal preparations should be encouraged and public education on the good and bad effects of herbals need to be emphasized. Health care professionals should remain vigilant for potential interactions between herbals and prescription medications.

## Authors’ contributions

All authors wrote the paper equally.

## Conflict of interests

The authors declared no competing interests.

## Ethical considerations

Ethical issues (including plagiarism, data fabrication, double publication) have been completely observed by the authors.

## Funding/Support

None.
